# Record linkage to obtain birth outcomes for the evaluation of screening biomarkers in pregnancy: a feasibility study

**DOI:** 10.1186/1471-2288-9-48

**Published:** 2009-07-09

**Authors:** Samantha J Lain, Charles S Algert, Vitomir Tasevski, Jonathan M Morris, Christine L Roberts

**Affiliations:** 1 Perinatal Research, Kolling Institute of Medical Research, University of Sydney, Syndey, NSW, Australia; 2 Fetal Maternal Medicine (PaLMs), Royal North Shore Hospital, St Leonards, NSW, Australia

## Abstract

**Background:**

Linking population health data to pathology data is a new approach for the evaluation of predictive tests that is potentially more efficient, feasible and efficacious than current methods. Studies evaluating the use of first trimester maternal serum levels as predictors of complications in pregnancy have mostly relied on resource intensive methods such as prospective data collection or retrospective chart review. The aim of this pilot study is to demonstrate that record-linkage between a pathology database and routinely collected population health data sets provides follow-up on patient outcomes that is as effective as more traditional and resource-intensive methods. As a specific example, we evaluate maternal serum levels of PAPP-A and free *β*-hCG as predictors of adverse pregnancy outcomes, and compare our results with those of prospective studies.

**Methods:**

Maternal serum levels of PAPP-A and free *β*-hCG for 1882 women randomly selected from a pathology database in New South Wales (NSW) were linked to routinely collected birth and hospital databases. Crude relative risks were calculated to investigate the association between low levels (multiples of the median ≤ 5^th ^percentile) of PAPP-A or free *β*-hCG and the outcomes of preterm delivery (<37 weeks), small for gestational age (<10^th ^percentile), fetal loss and stillbirth.

**Results:**

Using only full name, sex and date of birth for record linkage, pregnancy outcomes were available for 1681 (89.3%) of women included in the study. Low levels of PAPP-A had a stronger association with adverse pregnancy outcomes than a low level of free *β*-hCG which is consistent with results in published studies. The relative risk of having a preterm birth with a low maternal serum PAPP-A level was 3.44 (95% CI 1.96–6.10) and a low free *β*-hCG level was 1.31 (95% CI 0.55–6.16).

**Conclusion:**

This study provides data to support the use of record linkage for outcome ascertainment in studies evaluating predictive tests. Linkage proportions are likely to increase if more personal identifiers are available. This method of follow-up is a cost-efficient technique and can now be applied to a larger cohort of women.

## Background

Routinely collected data or population-health datasets (PHDS) have been used to address key issues in health. The population coverage and accessibility make PHDS an attractive and cost effective resource for research allowing description of the total burden of disease in the population, assessment of risk factors and casual pathways[1] and investigation of rare outcomes[2]. Linkage of population health data to pathology data is a new approach for evaluating predictive tests that is potentially more efficient and efficacious than current methods. Traditional methods for evaluation of predictive tests require evaluation in a representative spectrum of patients, and complete follow-up.[3] Such studies are often performed prospectively and require considerable resources. Successful linkage of pathology databases with those that routinely report health outcomes could provide a powerful and important tool for the evaluation of the clinical usefulness of a range of newly discovered biomarkers.

In Australia, antenatal screening for fetal anomalies, such as Down syndrome, has occurred since the 1980s[4,5]. The current screening test for Down syndrome combines first trimester maternal serum levels of pregnancy-associated plasma protein-A (PAPP-A) and free beta-human chorionic gonadotropin (free *β*-hCG) with ultrasound fetal nuchal translucency (NT) measurements and maternal age. An English study estimated that the detection rate with this type of screening is almost 90%[6].

There has been interest in the use of first trimester maternal serum biomarkers to predict other adverse pregnancy outcomes. Low levels of PAPP-A have been found to be associated with fetal growth restriction or low birth weight [7-14], preterm delivery[7-11,15] and fetal loss or stillbirth[7,9,12,15]. Studies investigating free *β*-hCG and adverse pregnancy outcomes have had mixed results[7,8,11,16].

The aim of this pilot study is to demonstrate that record-linkage between a pathology database and routinely collected population health data sets provides follow-up on patient outcomes that is as effective as more traditional and resource-intensive methods. As a specific example, we evaluate maternal serum levels of PAPP-A and free *β*-hCG as predictors of adverse pregnancy outcomes, and compare our results with those of previous studies.

## Methods

The dataset used in this analysis was built by linking records from three separate sources. The Pacific Laboratory Medicine Services (PaLMs) is a business unit of a network of northern Sydney public hospitals and analyses serum samples collected from across New South Wales (NSW), including both metropolitan Sydney and regional areas. The Midwives' Data Collection (MDC) is a legislated surveillance system of all births in NSW, reported by the attending midwife or doctor. The Admitted Patient Data Collection (APDC) has records of all hospital discharges in NSW (public and private), up to 55 diagnosis fields for each admission coded according to the 10th revision of the International Classification of Diseases – Australian Modification (ICD10-AM). Hospital records are available for liveborn infants, as well for mothers' antenatal and delivery admissions. MDC and APDC records were available for deliveries and hospital discharges up to 31 December 2006.

The initial study sample was randomly selected from blood tests collected for first trimester Down syndrome screening and analysed at the PaLMs laboratory, with the exclusion of known twin gestations. Both PAPP-A and free *β*-hCG were reported for each pregnancy. The initial sample included 2000 randomly selected tests collected between 1 January and 30 June 2006, subsequently reduced to 1882 women who were expected to deliver before 31 December 2006.

The record linkage for the study was conducted by an independent body (the NSW Centre for Health Record Linkage [CHeReL: http://www.cherel.org.au]), in accordance with NSW privacy guidelines and subject to ethics approval. Because there are no unique patient identifiers in use in the NSW health care system, probabilistic matching of personal identifiers (such as full name, date of birth, sex, address, hospital code and hospital record number) is used for linkage. Probabilistic record linkage involves the calculation of linkage probability weights, adjusting for data entry errors, incomplete and missing data, and has been described in detail elsewhere.[17,18] Once linked, personal identifiers are removed and a unique project person ID number is assigned to each record. Based on this linkage, data custodians of the relevant datasets provide health data (no identifiers) to researchers ensuring researchers at no time have access to identifying personal information and the risk to personal privacy is minimised.

For this pilot study, only mother's full name, sex and date of birth were available for linking laboratory records to pregnancy outcome data in the MDC and APDC. For pregnancies where multiple matches were possible (i.e. common names), no definite link to an outcome record could be established. This study was approved by the NSW Population and Health Services Research Ethics Committee.

### PAPP-A and free β-hCG measurement

Samples collected from regional centres were frozen prior to transport. An Immulite 2000 immuno-analyser (Siemens Healthcare Diagnostics) was used to measure serum levels of PAPP-A and free β-hCG. Both PAPP-A and free β-hCG results are reported as multiples of the median (MoM) corrected for gestational age and maternal weight, where provided, at sample collection. Corrections are based on the distributions of PAPP-A and free β-hCG in the laboratory's obstetric population. Gestational age was usually estimated by ultrasound, but date of last menstrual period was used where an ultrasound estimate was not recorded. For comparison with results from other studies, a cut-off of ≤ 5^th ^percentile MoM was used to define low PAPP-A and low free β-hCG results. This has been the most commonly used threshold in previous studies of these biomarkers as predictors of prematurity and intrauterine growth restriction.

### Pregnancy outcomes

Outcomes were selected that could be compared with previously published studies. The primary outcomes were preterm birth (birth at 24–36 weeks gestation) and small-for-gestational-age (SGA). SGA births were categorised as <10^th ^birthweight percentile for gestational age, by sex, using Australian standard birthweight percentiles[19]. Secondary outcomes were fetal loss (death before 24 weeks) and stillbirth (death *in utero *at ≥ 24 weeks). A composite outcome comprising fetal loss, stillbirth, birth at ≤ 37 weeks gestation or SGA birth was also analysed, although this was not directly comparable to results from other studies.

### Statistical analysis

The *t *test for unpaired samples was used to test for differences between linked and unlinked pregnancies for mean values of maternal factors or blood test results. A chi-square test was used to test for difference in proportions. McNemar's test was used to test for a difference in sensitivity for low PAPP-A versus low free β-hCG in the linked pregnancies. Sensitivity was defined as the percentage of pregnancies that resulted in an adverse outcome which were flagged as "at risk" by a PAPP-A or free β-hCG result ≤ 5^th ^percentile. Positive predictive value (PPV) was defined as the percentage of pregnancies that had an adverse outcome out of all pregnancies which tested "positive" for risk (had a PAPP-A or free β-hCG result ≤ 5^th ^percentile). The associations between low values of PAPP-A and free β-hCG and the pregnancy outcomes are presented as crude risk ratios (RR) with 95% confidence intervals (95% CI).

## Results

Of the 1882 women who had an expected delivery date in 2006, linkage to a pregnancy outcome record was established for 1681 (89.3%), leaving 201 (10.7%) with no outcome data. A further 16 twin pregnancies, 11 medical terminations less than 20 weeks gestation and 4 infants diagnosed with aneuploidies were excluded, so that 1650 pregnancies were included in the analysis. The median gestational age at which samples were collected was 12^+1 ^weeks, with a range of 10^+0 ^to 13^+6 ^weeks. Table 1 shows a comparison of days gestation at sample collection, maternal weight, PAPP-A and free β-hCG values, for the 1650 pregnancies in the analysis and the 201 pregnancies with no outcome data. There was no evidence that the unlinked pregnancies were different in regards to their PAPP-A and free β-hCG results. For instance, P = 0.28 for the test of a difference in proportion falling below the ≤ 5th percentile PAPP-A cutoff (Table 1)

**Table 1:** Maternal factors and biomarker results for pregnancies that did and did not link to a pregnancy outcome record

Maternal factor at time of screen test	Pregnancies linked to an outcome N = 1650*	Unlinked pregnancies N = 201	Test of difference
	*mean (sd)*	*mean (sd)*	
Days gestation	84.9 (6.0)	85.7 (5.9)	P = 0.08
Age (years)	32.6 (4.7)	32.0 (4.8)	P = 0.13
Weight (kg)	67.1 (14.4)^†^	65.2 (14.4)^†^	P = 0.13
PAPP-A (MoM)	1.19 (0.75)	1.24 (0.80)	P = 0.49
free β-hCG (MoM)	1.24 (0.81)	1.18 (0.74)	P = 0.26
			
	≤ *5*^*th *^*percentile cutoff *(%)	≤ *5*^*th*^*percentile cutoff *(%)	
PAPP-A (MoM)	5.3	3.5	P = 0.28
free β-hCG (MoM)	5.2	5.0	P = 0.92

* multiple gestations, aneuploidies and pregnancies resulting in medical abortions excluded

† (records with missing weight: linked n = 299, unlinked n = 51)

Seventeen of the 1650 pregnancies ended in a fetal loss at <24 weeks, the remaining 1633 pregnancies linked to a delivery record at ≥ 24 weeks gestation. Sixteen infants were stillborn, with a gestational age range from 24 to 41 weeks. Among the 1617 livebirths, two (0.1%) were at <28 weeks, 16 (1.0%) at 28–33 weeks, 52 (3.2%) at 34–36 weeks and 1547 (95.7%) at ≥ 37 weeks.

The relative risks of adverse outcomes for pregnancies with a PAPP-A MoM ≤ 5^th ^percentile and a free β-hCG MoM ≤ 5^th ^percentile are shown in Table 2. In this study population, the 5^th ^percentile MoM values were 0.38 of the median for PAPP-A and 0.40 of the median for free β-hCG. PAPP-A had a stronger association with all four adverse outcomes than free β-hCG. This was most evident for preterm birth, where a low PAPP-A result had RR = 3.44 (95% CI 1.94, 6.10) while free β-hCG did not have a statistically significant association.

**Table 2:** PAPP-A and free β-hCG results and pregnancy outcomes

Pregnancy outcome	PAPP-A ≤ 5^th ^percentile (MoM = 0.38)		free β-hCG ≤ 5^th ^percentile (MoM = 0.41)	
	n/N	RR (95% CI)*	n/N	RR (95% CI)*
*Gestational age*				
Preterm birth 24–36 weeks^†^	12/78	3.44 (1.94, 6.10)	5/78	1.31 (0.55, 3.16)
Birth at ≥ 37 weeks^†^	70/1555	1.0 (referent)	76/1555	1.0 (referent)
				
*Mortality outcome*				
Fetal loss <24 weeks	5/17	7.41 (2.67, 20.6)	4/17	5.61 (1.87, 16.8)
Stillbirth ≥ 24 weeks	0/16	not calculated	0/16	not calculated
Livebirth	82/1617	1.0 (referent)	81/1617	1.0 (referent)
				
*Birthweight for gestational age*				
SGA^† ^(<10^th ^percentile)	13/131	2.08 (1.23, 3.53)	12/131	1.93 (1.11, 3.34)
≥ 10^th ^percentile	69/1499	1.0 (referent)	69/1499	1.0 (referent)
				
*Composite outcome*				
Fetal loss, stillbirth, birth <37 wks or SGA^††^	27/228	2.41 (1.72, 3.39)	20/228	1.77 (1.18, 2.65)
Livebirth ≥ 10^th ^percentile	60/1422	1.0 (referent)	65/1422	1.0 (referent)

* relative risk of respective pregnancy outcome given a PAPP-A or free *β*-hCG ≤ 5^th ^percentile

† small for gestational age, includes stillbirths

†† small for gestational age

The stronger association of PAPP-A with adverse outcomes was also reflected in their respective sensitivities for those outcomes, although the differences in sensitivity were not statistically significant. For preterm birth, PAPP-A had a sensitivity of 15.4% versus 6.4% for free β-hCG (P = 0.14); for SGA birth, PAPP-A had a sensitivity of 9.9% versus 9.2% for free β-hCG (P = 0.99). The positive predictive value (PPV) of PAPP-A for preterm birth was 14.6% and for free β-hCG was 6.2% (P = 0.08). For the composite outcome including preterm birth, fetal loss, stillbirth and SGA birth, PAPP-A had a PPV of 31.0% and free β-hCG had a PPV of 25.3% (P = 0.27).

## Discussion

This study demonstrates the feasibility of using record-linkage of a pathology database with existing population health datasets to ascertain pregnancy outcomes for evaluating predictive tests. We found that a low level of PAPP-A has a stronger association to adverse pregnancy outcomes than a low level of free *β*-hCG which is consistent with previous studies[7-10,20-22]. This study provides data to support the use of such a cost-efficient technique which can now be applied to assessment of other potential pregnancy screening factors (such as levels of the VEGF family of proteins and their receptors in first trimester serum samples) in a larger cohort of women.

Prospective studies evaluating the diagnostic value of first trimester maternal serum levels have shown a range of risk estimates between low levels of PAPP-A or free *β*-hCG and adverse pregnancy outcomes, as summarised in Tables 3A and 3B. Although the results of our study show a stronger association between PAPP-A and preterm delivery or fetal loss than some of the larger published studies, the 95% confidence intervals of all results overlap. Our study sample size (initially 2000 lab records, ultimately 1,650 women) was selected on the basis of being large enough to show consistency with results published in other studies, not to demonstrate a clinical benefit of using these particular maternal biochemical markers as predictors of adverse outcomes in pregnancy. High levels of free *β*-hCG in the second trimester have been associated with preterm delivery[23] and fetal death[24] however in this study we have focused on the predictive value of low levels of biochemical markers in the first trimester.

**Table 3:** Summary of results from this study and relevant published studies of PAPP-A and adverse pregnancy outcomes and Free *β*-hCG and adverse pregnancy outcomes

*A*. PAPP-A and adverse pregnancy outcomes				
***Outcome***	**PAPP-A *Study***	***Population (N)***	***MoM cut-off***	OR/RR (95% CI)*

Preterm delivery <37 weeks	This study	1,650	≤ 5^th ^percentile (0.38 MoM)	3.44 (1.94–6.10)
	Barrett *et al *2008[7]	10,273	≤ 0.3 MoM	2.9 (2.0–4.2)
	Smith *et al *2002[11]	8,839	≤ 5^th ^percentile (0.40 MoM)	2.34 (1.75–3.12)
	Dugoff *et al *2004[9]	34,271	≤ 5^th ^percentile (0.42 MoM)	1.73 (1.47–2.04)^†^
	Ong *et al *2000[8]	5,297	≤ 5^th ^percentile	1.52 (0.91–2.55)
				
Birth weight ≤ 10^th ^percentile for gestational age	This study	1,650	≤ 5^th ^percentile (0.38 MoM)	2.08 (1.23–3.53)
	Krantz *et al *2004[10]	8,012	≤ 5^th ^percentile (0.45 MoM)	2.7 (1.9–3.9)^††^
	Tul *et al *2003[13]	1,136	≤ 0.5 MoM	2.51 (1.30–4.83)
	Dugoff *et al *2004[9]	34,271	≤ 5^th ^percentile (0.42 MoM)	2.47 (2.16–2.81)^†^
	Ong *et al *2000[8]	5,297	≤ 5^th ^percentile	1.53 (1.08–2.17)
				
Fetal loss <24 weeks	This study	1,650	≤ 5^th ^percentile (0.38 MoM)	7.41 (2.67–20.6)
	Spencer *et al *2006[21]	54,722	≤ 5^th ^percentile (0.42 MoM)	3.25
	Dugoff *et al *2004[9]	34,271	≤ 5^th ^percentile (0.42 MoM)	2.5 (1.76–3.56)^†^

***B*. Free *β*-hCG and adverse pregnancy outcomes**				

***Outcome***	**Free *β*-hCG *Study***	***Population (N)***	***MoM cut-off***	OR/RR (95% CI)*

Preterm delivery <37 weeks	This study	1,650	≤ 5^th ^percentile (0.41 MoM)	1.31 (0.55–3.16)
	Barrett *et al *2008[7]	10,273	≤ 0.3 MoM	2.0 (1.3–3.1)
	Ong *et al *2000[8]	5,297	≤ 5^th ^percentile	1.29 (0.71–2.34)
				
Birth weight ≤ 10^th ^percentile for gestational age	This study	1,650	≤ 5^th ^percentile (0.41 MoM)	1.93 (1.11–3.34)
	Ong *et al *2000[8]	5,297	≤ 5^th ^percentile	1.38 (0.93–2.03)
	Krantz *et al *2004[10]	8,012	≤ 5^th ^percentile (0.45 MoM)	1.3 (0.8–2.0)^††^
				
Fetal loss <24 weeks	This study	1,650	≤ 5^th ^percentile (0.41 MoM)	5.61 (1.87–16.8)
	Spencer *et al *2006[21]	54,722	≤ 5^th ^percentile (0.41 MoM)	3.1

*The crude relative risk and 95% confidence intervals has been calculated from published results where possible

^†^OR adjusted for maternal age, maternal weight, parity, previous preterm pregnancy, threatened abortion, smoking, use of antihypertensive drugs

^††^OR adjusted for maternal age, maternal weight, smoking, ethnicity

Sensitivities and predictive values reported in previous studies are also comparable to our results. Of those studies that used PAPP-A cut-offs less than the 5^th ^percentile, sensitivities for small for gestational age infants ranged from 7.8%[8] to 11.4%[22] compared to our result of 9.9% and positive predictive values ranged from 14.1%[10] to 16.7%[22] compared to our PPV of 15.8%. For preterm birth, the sensitivity for PAPP-A lower than the 5^th ^percentile estimated from our results (15.5%) is higher than reported in other studies (7.8% to 8.5%)[8,9] however Kwik and Morris[14] demonstrated a sensitivity of 17.7% for a PAPP-A cut-off at less than 0.5 MoM.

Rigorous evaluation of predictive serum tests traditionally requires the assay to be performed in a prospective cohort, representative of those in whom the test will be used in clinical practice. In addition, patients and carers are blinded to the test result and complete follow-up to the endpoint of interest is required.[3] The follow-up of women to ascertain pregnancy outcomes is often obtained by interview with the study participants or review of medical records. Outcomes derived from interview are subject to patient recall bias[25,26] and possibly to interviewer bias (if exposure is not blinded). Follow-up of pregnant women is also difficult as a high proportion of them are likely to have moved house during their pregnancy (around 20%)[27] and this non-response bias would affect the validity and generalisability of the result. Medical record review is time consuming, and both methods of follow-up are expensive. Ascertainment of pregnancy outcomes by linkage to existing population health databases can overcome some of these constraints, the costs of linkage in this study was AUD8500. In contrast, the largest prospective study in pregnancy http://www.scopestudy.net/news.aspx has been recruiting internationally for over 4 years and by April 2009 had enrolled over 4000 women. This study involves repeated collection of biological samples (including serum) during pregnancy and, to date has had AUD11.4M of funds allocated with the aim of recruiting 10,000 women.

Using record linkage to attain outcome data can reduce the risk to personal privacy for participants in research studies[28]. In our study, the processes of record linkage and data analysis were separated: use of identifiable data was restricted to the record linkage, the linkage was conducted by someone not involved in the research, and access only to anonymised data were available to the investigators, maximising protection of participants' privacy. Women participating in the study did not need to be contacted again minimising the risk of harm to participants, especially those that have experienced a negative outcome such as a fetal loss or infant death. A further advantage is that the linkage to outcomes is done while blinded to exposure, in this instance the laboratory results.

In this study almost 90% of women could be linked to pregnancy outcome data. This linkage rate is less than in other studies[7], however linkage was only performed on three identifying fields. A Scottish study using probabilistic linkage of maternal surname, first and second initial, date of birth and postcode, where available, reported a linkage rate of 81%[29]. With more identifiers available the linkage rate in this study should be improved considerably. Using matching variables including full name, address (including phonetic coding), postcode, country of birth, hospital code and medical record number results in over 98% of records linked[30].

The use of PHDS to determine pregnancy outcomes has its limitations. Only outcomes that are routinely collected can be evaluated and the quality of results is dependent on the accuracy and completeness of data recorded in the population datasets. Validation studies that compare PHDS with corresponding medical records have shown the outcomes used in this study to be reported reliably in PHDS. The hospital data reporting of preterm birth has a sensitivity of 76% [31] and in the MDC reporting of gestational age has 85% exact agreement with medical records[32]. Identifying outcomes from more than one dataset, such as from both the MDC and the APDC, has been shown to improve case ascertainment.[33] The timeliness of the availability of routinely collected data may be a limitation. Population data are collected all year and then typically closed out at the end of the financial or calendar year. The data must then be cleaned before linkage can be done; in total this process can take a number of years. Despite these limitations data linkage is a powerful method to investigate possible associations that may be worth pursuing.

## Conclusion

This feasibility study demonstrates that predictive tests can be evaluated by linking pathology databases with routinely collected population health databases that report pregnancy outcomes. This method of outcome ascertainment is less expensive than other methods of follow-up, minimises potential biases associated with outcome collection and loss to follow up and reduces the risk to patient privacy. Record linkage will provide a powerful and important tool for the evaluation of the clinical usefulness of a range of newly discovered biomarkers with major implications for translating research findings into clinical practice, not only in pregnancy and newborn health but also longer term health outcomes.

## Competing interests

The authors declare that they have no competing interests.

## Authors' contributions

CR, JM and VT originated the idea for and design of this study. CA and SL performed data analysis and prepared the manuscript. All authors were involved in the drafting and revision of the manuscript and approved the final manuscript.

## Pre-publication history

The pre-publication history for this paper can be accessed here:

http://www.biomedcentral.com/1471-2288/9/48/prepub
